# Genetic variation in the invasive avian parasite, *Philornis downsi *(Diptera, Muscidae) on the Galápagos archipelago

**DOI:** 10.1186/1472-6785-8-13

**Published:** 2008-07-31

**Authors:** Rachael Y Dudaniec, Michael G Gardner, Steve Donnellan, Sonia Kleindorfer

**Affiliations:** 1 School of Biological Sciences, Flinders University GPO Box 2100, Adelaide, South Australia 5001, Australia; 2 Centre for Evolutionary Biology and Biodiversity, University of Adelaide, South Australia 5005, Australia

## Abstract

**Background:**

Understanding the dispersal and genetic structure of invasive insects across islands is important for designing management plans that are appropriate at spatial and temporal scales. For invasive parasites, population dynamics are largely determined by the distribution and density of their host species. The introduced parasitic fly, *Philornis downsi*, parasitises nestlings of endemic birds on all major islands of the Galápagos archipelago. The fly's high mortality and fitness impacts are of conservation concern for vulnerable and declining species of Darwin's finches. Using microsatellite data in Bayesian clustering and landscape genetic analyses, we examine gene flow and dispersal in *P. downsi *between three islands and across habitats (highlands, lowlands) and examine for the presence of population bottlenecks. We also examine variation at the mitochondrial gene *CO1 *across islands to establish if cryptic species were present.

**Results:**

Both the mitochondrial and microsatellite data were consistent with there being a single species across islands. We found low genetic differentiation between islands and strong evidence for inter-island gene flow, or shared recent ancestry among individuals. Landscape genetic analysis identified two genetic clusters: one encompassing Santa Cruz and Isabela, and one on Floreana Island. There was no evidence of genetic differentiation between habitats and molecular variance was mainly attributable to within individuals. The combined *P. downsi *population was found to have undergone a population bottleneck.

**Conclusion:**

*Philornis downsi *populations have high connectivity within and between islands, with low levels of genetic differentiation between Floreana and the other two islands examined. The genetic bottleneck found across islands suggests there was a small founding population or few introduction events of *P. downsi*. The high dispersal capacity and wide habitat use of *P. downsi *highlights the significant threat that this parasite poses to the Galápagos avifauna. Our findings are relevant for assessing the viability of methods to control *P. downsi *on Galápagos, such as the sterile insect technique.

## Background

Biological invasions threaten biodiversity and ecosystem function, with pronounced negative effects on islands in particular [1-3]. Genetic studies of invasive species can identify the adaptive potential of invaders to deal with new environmental conditions [4] or help to predict evolutionary responses to management practices (e.g. pesticides, biological control agents) [5]. Population bottlenecks affect many invasive species because they frequently experience founding effects that reduce genetic variability, but paradoxically, invasive species still manage to successfully establish and adapt to new environments [6]. However, the effects of bottlenecks may be countered by the occurrence of multiple introductions, high reproductive rates, and subsequent migration between locally bottlenecked populations that are genetically differentiated [7].

For invasive arthropod parasites, these factors are inextricably linked with the distribution, genetics, and behaviour of host species [8-10]. The recent integration of molecular ecology with parasitology has provided a path for answering a number of questions concerning the genetic structure of parasite populations, which can uncover a wealth of information regarding ecological and evolutionary processes for invasive parasites [10]. Highly variable multilocus genotypes are particularly suited to analyses of non-equilibrium or bottlenecked populations because they provide adequate variation for assessing recent gene flow and identifying migrants [11].

The introduced fly, *Philornis downsi*, is an avian ectoparasite that is considered to be a serious threat to the persistence of endemic finch populations on the Galápagos Islands [12-14]. Recently, *P. downsi *was given the highest risk ranking affecting endemic fauna in the Galápagos archipelago [3]. Other pathogens affecting Galápagos birds such as avian pox virus [15] and intestinal protozoans [16] are of less concern, but may also cause high fitness impacts under certain conditions. The fly was first formally identified from Darwin finch nests in 1997 and has since been found on 11 of 13 major islands in nests of 14 endemic species [12,13]. However, *P. downsi *colonised the islands at least 40 years ago, as the fly was identified recently from collections made in 1964 [13]. The blood-feeding larvae of *P. downsi *are associated with 62–100% nestling mortality in Darwin's finches [12,14,17], as well as physiological costs [18] and reduced growth rates in nestlings [14]. Little is known about the ecology and biology of *Philornis *flies and the dispersal behaviour and population genetics of the genus *Philornis *or of any other myiasis-causing parasite of birds [reviewed in [13]].

One potential control method to eradicate *P. downsi *is the sterile insect technique (SIT), which is renowned for its effectiveness at eradicating or suppressing fruit fly and screw-worm fly populations across the globe [19,20]. SIT involves the large-scale release of laboratory-reared sterile male (and/or female) flies that eventually suppress fly populations by reducing population fecundity [reviewed in [20]]. SIT requires a thorough understanding of the reproductive ecology and population dynamics of the target species. The effectiveness of SIT is affected by the occurrence of genetically divergent 'strains' of the target species across the geographic area under control because this is detrimental to the mating success of sterile flies [19,21,22]. Specifically, high genetic divergence may reflect differences in behaviour and/or morphological characteristics that result in mating incompatibility among populations of the target species [21,23]. Thus, target populations that show low genetic divergence are not likely to show reproductive isolation and influence the success of a particular sterile strain.

The Galápagos archipelago offers a unique system to examine the population genetics of an introduced avian parasite that causes severe fitness costs and that is still within a relatively early phase of invasion. We collected parasites in 2004, 2005 and 2006 from three islands of the Galápagos. Using mitochondrial data, we firstly determine whether the three island populations from which we sampled are of the one fly species. We then use microsatellite data to examine gene flow within and among islands to: (1) determine whether dispersal and genetic divergence are occurring among islands and between habitats within islands (wet highlands, arid lowlands), (2) determine the presence of population bottlenecks resulting from the invasion process, and (3) determine whether inter-island genetic differentiation may be of concern to the potential success of an archipelago-wide SIT program for controlling *P. downsi*.

## Methods

### Study species

*Philornis downsi *(family Muscidae; subfamily Azeliinae; tribe Reinwardtiini) is a semi-haematophagous obligate avian parasite in its three larval stages, whereas adult flies are non-parasitic and feed on organic matter [13]. Adults lay eggs inside the nares of newly hatched nestlings (usually at one to three days old), which hatch into first instar larvae [17,24]. Second and third instar larvae attach externally and feed on nestling blood and tissues over four to six days [13]. Most larvae of *P. downsi *appear to reach their third instar phase at the time of host fledging. The larvae pupariate at the base of the nesting material and remain for approximately two weeks before emerging as adult flies [13,25].

### Study area and sample collection

*Philornis downsi *were collected from three islands of the Galápagos: Santa Cruz (986 km^2^; 0° 37'S, 90° 21'W), Floreana (173 km^2^, 1° 28'S, 90° 48'W), and Isabela (4588 km^2^, 0° 58'S, 90° 58'W). Fly samples were collected from nests during the January to March finch breeding season in 2004, 2005 and 2006 from two contrasting habitats, the arid lowlands (0–100 m asl) and the humid highlands (300–600 m asl) (Table 1) [see also [26,27]]. The lowlands are characterised by low rainfall, and are dominated by the trees *Acacia macracantha*, *Bursera graveolens*, *Croton scouleri*, *Opuntia *spp., *Pisonia floribunda*, and *Zanthoxylum fagara *[12]. In contrast, the highlands have much higher rainfall [28,29], abundant moss and lichen, and are dominated by the endemic tree *Scalesia pedunculata*, or *S. cordata *(Asteraceae) on Isabela Island.

**Table 1:** Sample sizes of bird nests and *P. downsi *individuals.

		**# Bird nests sampled ** **for *P. downsi***		**# Individuals ** **analysed**	
		
**Year**	**Island**	Highland	Lowland	Highland	Lowland
2004	Santa Cruz	3	18	11	51
	Floreana	1	4	1	9
	Isabela	2	1	7	2
2005	Floreana	11	-	28	-
2006	Santa Cruz	-	2	-	5
	Floreana	11	10	30	14

	**Total**	**28**	**35**	**77**	**81**

The number of nests and the number of individuals analysed (following construction of dataset comprising unrelated individuals) for each island and habitat (highland/lowland) across three islands, Santa Cruz, Floreana, and Isabela in 2004, 2005 and 2006.

**Figure 1:**
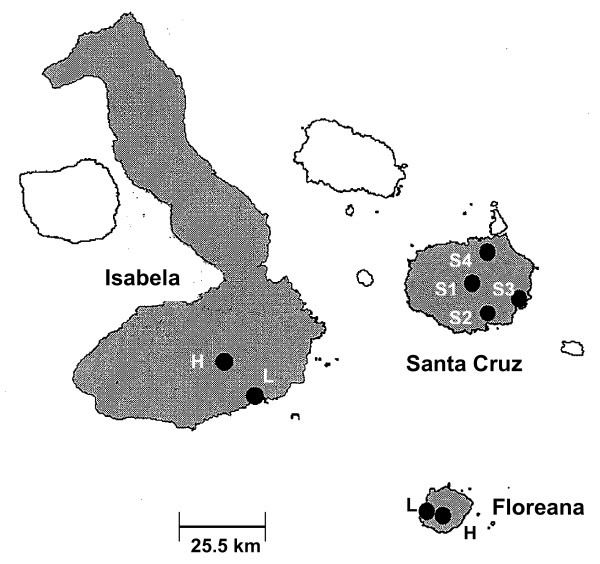
**Map of Santa Cruz, Floreana, and Isabela Islands with sampling locations**. Sampling sites on Santa Cruz: S1 = highlands; S2 = lowlands, Punta Estrada; S3 = lowlands, Garrapatero; S4 = lowlands, Itabaca. On Floreana and Isabela, one site each in the lowlands (L) and highlands (H) are indicated.

We sampled from one site in each habitat on both Floreana (lowlands, adjacent to the town of Puerto Velasco Ibarra: 1° 16'S, 90° 29'W; highlands, base of Cerro Pajas: 1° 17'S, 090° 27'W) (Figure 1) and Isabela (lowlands: adjacent to town of Puerto Villamil: 0° 57'S, 91° 00'W; highlands: 0° 50'S, 91° 01'W), while on Santa Cruz we sampled from three sites in the lowlands: (1) Garrapatero: 0° 39'S, 90° 28'W; (2) Itabaca: 0° 29'S, 90° 17'W; (3) Punta Estrada, near Puerto Ayora: 0 ° 45'S, 90° 18'W, and one site in the highlands (Los Gemelos: 0° 37'S, 90° 22'W) (Figure 1). All sample sites were approximately 2000–4000 m^2^, except for the highland site on Isabela, where our sample site was only 100 m^2 ^because habitat fragmentation has reduced the *Scalesia *forest to small remnant patches. The distance between highland and lowland sites was much shorter on Floreana (3–5 km) than on Santa Cruz and Isabela (both 15–25 km), while on Santa Cruz, the distance between all four sites (1 highland, 3 lowland) varied between 15 and 27 km. Data were obtained from all three islands in 2004, from just Floreana in 2005, and from Santa Cruz and Floreana in 2006 (Table 1).

For the purpose of our study, larvae, puparia and puparia cases were sampled from 64 bird nests of five Darwin finch species (*Geospiza fuliginosa*, n = 25, *Geospiza fortis*, n = 15, *Camarhynchus parvulus*, n = 3, *Camarhynchus pauper*, n = 4; *Cactospiza pallida*, n = 1), while one nest was opportunistically sampled from each of the Galápagos mockingbird (*Nesomimus parvulus*) and the yellow warbler (*Dendroica petechia aureola*). Fourteen recently fledged nests were sampled for *P. downsi *where the finch species was unknown. GPS coordinates were recorded at each nest location. Inactive nests were collected and sealed in individual plastic bags and later dismantled for counting of *P. downsi *individuals. All flies were immediately preserved in 95% ethanol.

### DNA extraction and microsatellite typing

DNA extraction was carried out using the salting out procedure described in [30] with the exception that all samples (3 mm^2 ^tissue from each individual) were homogenised and washed three times in 10 mm TRIS prior to digestion with Proteinase K to remove traces of ethanol, excess lipids, and other potential contaminants. Across all three islands, 1012 *P. downsi *individuals (larvae and pupae) were genotyped (Table 1) using eight microsatellite markers [31]: Pd1 [GenBank: EF608562] Pd2 [EF608556], Pd4 [EF608557], Pd6 [EF608564], Pd7 [EF608558], Pd8 [EF608555], Pd9 [EF608561], Pd10 [EF608563]. Multiplex PCR conditions were followed as described in Dudaniec et al. [31]. Samples were genotyped on an ABI 3730 capillary electrophoresis DNA analyser (Applied Biosystems). A fluorescently labeled size standard (GS500 (-250) LIZ) was run with the samples and alleles were scored using GENEMAPPER version 3.7 (Applied Biosystems). To minimise and estimate genotyping error, each run of the DNA analyser contained eight repeated samples and a control sample run each time. In total, this resulted in 70 individual samples (14.5% of all samples genotyped) being re-amplified and genotyped at least once.

### Mitochondrial DNA sequencing

An 822-bp region of the 3' end of the *CO1 *gene was amplified in five *P. downsi *individuals collected from Santa Cruz (1 highlands), Floreana (1 highlands, 1 lowlands), and Isabela (1 highlands, 1 lowlands). Samples were amplified using primers M202 (forwards, C1-J-1751 [32]) and M70 (reverse, UEA10 [33]). Amplifications were performed in 10× *Taq*Gold buffer, 25 mM MgCl_2_, 10 mM total dNTP's, 200 nM each primer, 0.2 U *Taq*Gold polymerase, and 10–50 ng DNA. Amplification conditions were an initial denaturation at 94°C for 9 min, followed by 34 cycles of 94°C for 45 s, 55°C for 45 s, 72°C for 1 min, with a final extension of 72°C for 6 min. Sequencing was performed using the ABI Prism™ Big Dye Terminator Cycle sequencing kit (Applied Biosystems) according to the manufacturer's instructions. Products were sequenced on ABI 3700 (version 3.7) automated DNA sequencers. SeqEd (version 1.0.3) (Applied Biosystems) was used to edit chromatogram files to determine bi-directional consensus sequences and to manually align sequences across samples.

### Allele frequencies and data set construction

We calculated allele frequencies using RELATEDNESS 5.0.8 [34] by randomly selecting one individual per sample (n = 64) to eliminate the possibility of including related individuals (a sample is defined as all *P. downsi *individuals collected from a single bird's nest). Exact tests were performed for each microsatellite locus to test deviation from Hardy-Weinberg equilibrium using GENEPOP[35]. All loci were in Hardy Weinberg equilibrium after sequential Bonferroni correction [36] and these allele frequencies were used for all further analyses. Genetic relatedness among *P. downsi *offspring within nests of Darwin's finches is low, and the individuals found within each nest are produced by up to approximately five ovipositing females that have each mated with between one and five males (as found by sib-ship reconstruction analysis by Dudaniec et al. in review). To eliminate the effect of sibs in the data, we selected unrelated individuals that were identified using the sib-ship reconstruction method implemented in the program COLONY 1.2 [37]. Each sample of *P. downsi *individuals taken from an independent bird nest was run in COLONY 1.2, which uses a maximum likelihood method that partitions individuals into pure full-sib families (i.e. monogamous female parent), or full-sib families nested within half-sib families (i.e. polyandrous female parent) using progeny genotypes without known parental genotypes [37,38]. Three runs were performed per sample with different random seed numbers (12, 80, and 243) to ensure data convergence, and a conservative error rate of 5% was implemented based on evidence from the re-genotyping of 70 individuals, in which genotyping error ranged from 0–5% across loci.

We selected one individual per reconstructed maternal family (i.e. one family = the offspring assigned to one putative female parent). In nested-half sib families (i.e. one mother, multiple fathers), individuals were only selected from full sib families with the largest number of members that had the highest posterior probability. Only individuals genotyped at all eight loci were included in the analysis and individuals were not sampled from families that contained Class I or Class II typing errors (identified by COLONY 1.2) [37]. These criteria resulted in a sample size of 158 individuals sampled from 63 bird nests (with between one and six unrelated individuals per sample) (Table 1).

To examine the probability that two randomly selected individuals from the same population will have the same multi-locus genotype, a Probability of Identity (PI) analysis was performed using GIMLET[39]. The output is a cumulative multi-locus PI value, estimated both with and without sample size correction. PI values were calculated for the dataset of 158 individuals using equations of unbiased PI, which assumes that individuals are unrelated, and PI for sibs, which assumes that all individuals are siblings [39].

### Inter-island genetic differentiation

Heterozygosity, and pairwise *F*st [40] was calculated to examine genetic differentiation between islands (Santa Cruz, Floreana, Isabela) using MICROSATELLITE ANALYSER (MSA) 4.05 [41]. Genotypic differentiation was tested between islands using option 3 with 10 0000 Markov chain iterations in GENEPOP[35]. P-values for multiple tests were adjusted using sequential Bonferroni correction [36]. The AMOVA method [42] was conducted in GENALEX version 6 [43] to partition the total genetic variation into three levels: among islands, among individuals, and within individuals using the Codom-genotypic distance calculation and 9999 permutations.

### Population bottleneck analysis

Recently colonised species may experience a population bottleneck, resulting in a reduction in the number of alleles and expected heterozygosity at polymorphic loci. However, alleles may be lost at a faster rate than the loss of heterozygosity, so observed heterozygosity is higher than the expected heterozygosity at equilibrium [44]. The program BOTTLENECK version 1.2.02 [45] was used to test for the presence of a recent population bottleneck for *P. downsi *by analysing within-population heterozygosity and allele frequency using the constructed dataset of individuals sampled from all islands. Both the stepwise mutation model (SMM) and two-phase model of mutation (TPM) were used, with the latter model being considered the most appropriate for microsatellites. The variance for the TPM was set at 5% and the proportion of SMM in TPM was set at 95% [46]. To determine differences in gene diversity across loci, the Wilcoxin sign-rank test was used as recommended for data sets with less than 20 loci (with 10 000 permutations) [45]. We also examined the allele frequency distribution in order to see whether it is approximately L-shaped (as expected under mutation-drift equilibrium) or not (indicating that a recent bottleneck has provoked a mode shift) as described in [47].

### Genetic structure among islands

For inferring genetic structure among the three sampled islands, we conducted two complementary individual-based Bayesian clustering analyses using STRUCTURE 2.1 [48] and the landscape genetics program GENELAND[49] without *a priori *knowledge of population units and limits. Both software packages implement a Bayesian clustering method that uses a MCMC technique to define the number of populations in a sample that are at Hardy Weinberg Equilibrium. The methods implemented in these two programs differ in that GENELAND determines the optimal number of populations or 'clusters' and then allocates individuals (probabilistically) to these clusters using geographic coordinates, whereas STRUCTURE carries out the allocation sequentially for different numbers of clusters, and then flags the number of clusters with the highest likelihood [50]. In STRUCTURE, the following run parameters were used: admixture without population information used, correlated allele frequency model, a burn-in period of 100 000 simulations followed by a run length of 1 million Markov Chain Monte Carlo (MCMC) simulations and three iterations for each number of potential clusters (defined as *k *= 1–5) to check for consistency of results. Estimation of *k *was taken to be the values of *k *with the highest Pr (X|*k*).

In contrast to STRUCTURE, the algorithm implemented in GENELAND[49] is considered to be a powerful clustering method under conditions of low genetic differentiation among populations [51,52]. The model infers genetic discontinuities between populations in space from multilocus genotypes obtained from geo-referenced individuals [49,53]. All individuals from the same sample (i.e. same bird nest) were allocated the same GPS coordinates. GPS coordinates were available for 57/63 nests (Santa Cruz: n = 18; Floreana: n = 36; Isabela: n = 3) (138 individuals in total). Samples for which GPS coordinates were missing were excluded from the analysis. To firstly infer the number of genetic clusters (*k*) in our data set, we used the Dirichlet model, which assumes independent allele frequencies with the following parameters: 1000 000 MCMC iterations, uncertainty attached to spatial coordinates = 0, variable number of populations = TRUE, minimum *k *= 1, maximum *k *= 5, and spatial information included in the model = TRUE. This procedure was performed three times to establish consistency of *k *across runs. The established *k *was then run five times to check the consistency of individual assignment to the inferred populations across runs. The same parameters were used but *k *was fixed at the modal number found in the first analyses. These five runs were post-processed (with a burn-in of 1000 × 100 iterations) to obtain posterior probabilities of population membership for each individual. Consistency of results across the five runs was checked visually.

Inferred populations were further examined for heterozygosity, allelic richness (corrected for sample size), observed (H_o_) and expected (H_e_) heterozygosity, inbreeding coefficients (*F*is), and genetic differentiation (estimated using *F*st) using FSTAT v. 2.9.3 [54].

## Results

### Mitochondrial sequencing

Sequences of the *CO1 *mitochondrial gene fragment in five individuals across islands showed almost no variation, with two individuals (one Santa Cruz highland and one Floreana highland) having an identical single nucleotide substitution (T-G). This supports the existence of one sampled species across the three islands.

### Genetic diversity and differentiation

Probability of identity (PI) analyses showed that the microsatellite loci had sufficient power and resolution for the analyses. The unbiased PI value was 1.333^-06^, and the PI for sibs was 2.610^-3^. This equates to one individual in approximately 751 880 having a non-unique genotype where individuals are unrelated (unbiased), and one individual in approximately 383 individuals having a non-unique genotype if all individuals are siblings.

The total number of alleles observed at each locus was as follows; Pd1 = 4; Pd2 = 3; Pd4 = 4; Pd6 = 5; Pd7 = 3; Pd8 = 4; Pd9 = 3; Pd10 = 3 (Table 2). There was significant genotypic differentiation across the three islands (Fisher's Exact method: *X*^2 ^= 72.75; df = 16; P < 0.001). Mean allelic richness across loci was almost identical on each island (Santa Cruz: 3.50; Floreana 3.63; Isabela: 3.5) and the range of observed heterozygosity across loci was also similar (Santa Cruz: 0.45–0.70; Floreana: 0.45–0.73; Isabela: 0.44–0.89). The number and size of alleles from each island population were the same at each locus with two exceptions: there was a unique allele at locus Pd6 on Isabela (allele frequency = 0.055), and at locus Pd7 on Floreana (allele frequency = 0.012), which were each detected only in a single individual. Pairwise *F*st analysis showed low, but significant levels of genetic differentiation between Santa Cruz and Floreana (*F*st = 0.02, P < 0.02) Isabela and Floreana (*F*st = 0.04, P < 0.02), but not between Santa Cruz and Isabela (*F*st = 0.01, P > 0.1). The low genetic differentiation found between islands was reflected in an AMOVA, which showed that just 2% of the molecular variance was attributable to variation among islands (sum of squares (SS) = 14.23; df = 2; variance components (V) = 0.052), 4% among individuals (SS = 413.06; df = 155; V = 0.133), and 94% within individuals (SS = 385.5; df = 158; V = 2.44).

**Table 2:** Allele frequencies for eight microsatellite loci in *P. downsi *within two genetic clusters.

		**Santa Cruz ** **and Isabela**	**Floreana**
*Pd1*	315	0.319588	0.219512
	323	0.134021	0.256098
	325	0.154639	0.109756
	327	0.391753	0.414634
			
*Pd2*	230	0.273196	0.268293
	236	0.582474	0.439024
	240	0.14433	0.292683
			
*Pd4*	252	0.35567	0.304878
	254	0.298969	0.353659
	256	0.036082	0.02439
	258	0.309278	0.317073
			
*Pd6*	241	0.391753	0.390244
	251	0.221649	0.280488
	259	na	0.012195
	261	0.139175	0.109756
	263	0.247423	0.207317
			
*Pd7*	201	0.371134	0.439024
	207	0.27835	0.268293
	210	0.340206	0.292683
	213	0.010309	na
			
*Pd8*	349	0.139175	0.195122
	353	0.087629	0.04878
	357	0.134021	0.097561
	365	0.639175	0.658537
			
*Pd9*	194	0.324742	0.414634
	200	0.469072	0.45122
	217	0.206186	0.134146
			
*Pd10*	189	0.216495	0.329268
	191	0.231959	0.134146
	193	0.551546	0.536585

Clusters were inferred using landscape genetic analysis: Cluster 1 = Santa Cruz and Isabela Island (n = 62); Cluster 2 = Floreana Island (n = 76); na = not applicable because allele was absent.

### Bottleneck analysis

**Figure 2:**
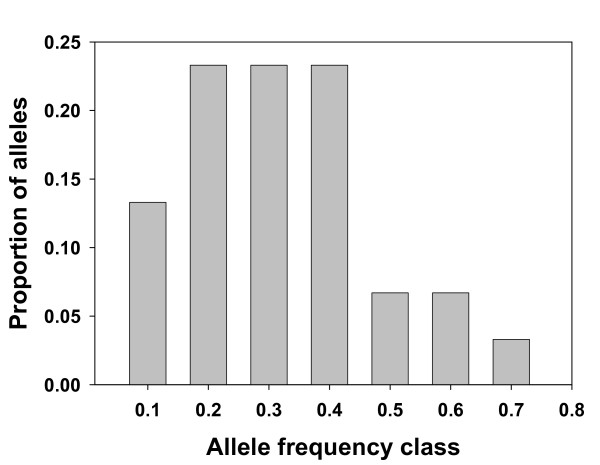
**Distribution of allele frequencies indicating a mode-shift**. Bars represent the proportion of alleles found in each allele frequency class. Deviation from an L-shaped distribution is indicative of a mode-shift in allele frequency due to a recent genetic bottleneck.

Combining individual from all islands (n = 158), a clear excess of heterozygosity (He) relative to the equilibrium heterozygosity (H_eq_) was observed, indicative of a population bottleneck under the TPM model (Wilcoxon sign-rank test; P < 0.01) and under the SMM model (P < 0.01). A mode-shift distortion in the distribution of allele frequencies was evident (Figure 2).

### Bayesian clustering analysis

**Figure 3:**
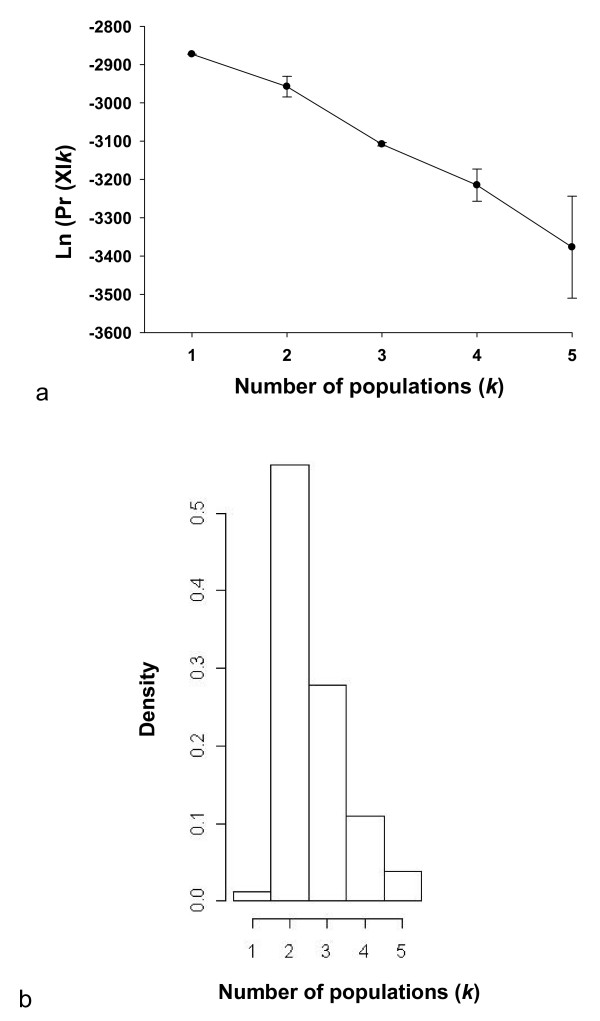
**Estimated number of populations from STRUCTURE (a) and GENELAND (b) analyses**. (a) Mean ( ± SD) probabilities of the data (LnPr [X|*k*]) over three replicate STRUCTURE runs plotted as a function of the putative number of clusters (*k*); (b) Posterior density distribution of the number of clusters estimated from GENELAND analysis in three replicates.

**Figure 4:**
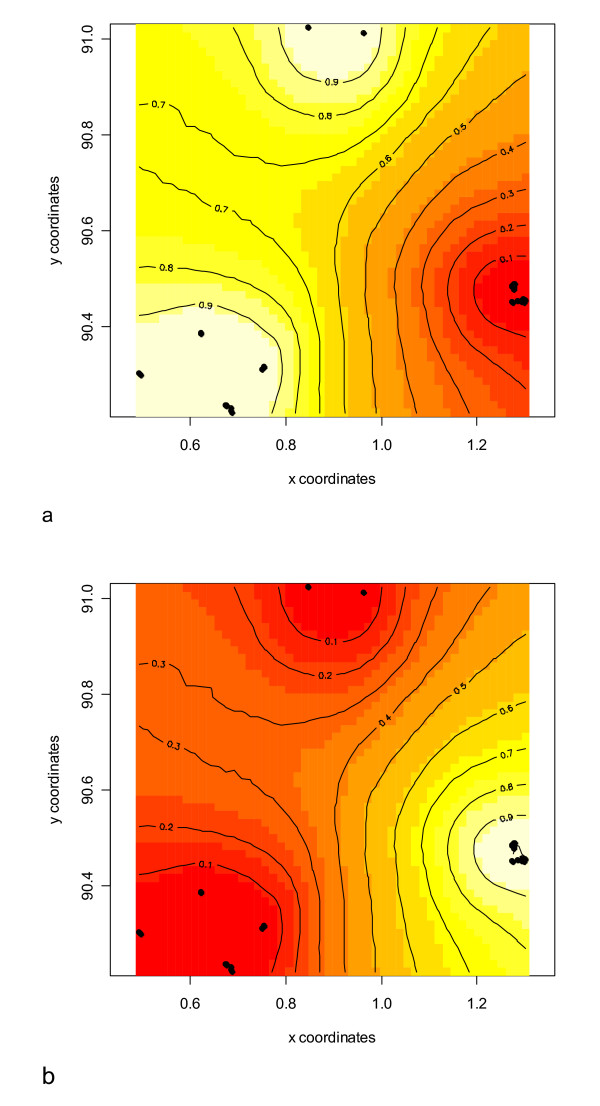
**Genetic assignment of *P. downsi *individuals across three islands using Bayesian clustering analysis**. Two genetic clusters are identified: (a) including all individuals from Santa Cruz (n = 62) (bottom left) and Isabela (n = 9) (centre top), and (b) all individuals from Floreana Island (n = 76) (bottom right). Black dots represent independent geographic sampling points (i.e. location of bird nests). Note that two geographic sampling points on Isabela Island were within 5 m of each other and are not distinguishable.

Individual-based cluster analysis using STRUCTURE[48] did not detect any genetic structuring in *P. downsi *collected across the three islands (Figure 3a), with individual assignment being evenly proportioned across variable numbers of *k*. This implies high levels of inter-island ancestry brought about by frequent dispersal and subsequent gene flow across the three islands sampled. However, when incorporating geographic coordinates of sampling locations into Bayesian analyses using GENELAND[49], two distinct genetic clusters were consistently found across runs (Figures 3b and 4). The first cluster includes all individuals sampled from Santa Cruz and Isabela Islands (n = 62), while the second cluster includes all individuals sampled from Floreana Island (n = 76). Assignment probabilities were between 0.98 and 1.0 across all individuals.

### Genetic diversity and differentiation among inferred clusters

The two clusters identified by GENELAND displayed comparable genetic diversity with regard to allelic richness and differed slightly in heterozygosity across loci (Tables 2 and 3). Although two clusters were detected, measures of genetic differentiation (*F*st) between them demonstrated the low divergence between individuals on Floreana Island and those on Santa Cruz and Isabela (*F*st = 0.024; 95% Confidence Interval (CI) = 0.014 – 0.034; P < 0.05). Tests of departure from HW equilibrium showed no significant deviation in either of the two clusters across all loci.

**Table 3:** Genetic variation at the eight microsatellite loci for the two *P. downsi *populations inferred from cluster analysis in GENELAND.

	**Santa Cruz and Isabela**			**Floreana**		
		
**Locus**	**A**	**H**_o_**/H**_e_	***F*is**	**A**	**Ho/He**	**Fis**
**Pd1**	4.0	0.63/0.71	0.038	4.0	0.71/0.71	0.091
**Pd2**	3.0	0.46/0.57	0.114	3.0	0.68/0.66	0.091
**Pd4**	4.0	0.67/0.69	0.105	3.0	0.64/0.67	0.037
**Pd6**	5.0	0.68/0.72	0.090	4.0	0.73/0.72	-0.012
**Pd7**	3.0	0.56/0.67	0.131	3.0	0.73/0.66	0.037
**Pd8**	4.0	0.48/0.55	0.023	4.0	0.61/0.52	0.034
**Pd9**	3.0	0.67/0.64	-0.083	3.0	0.68/0.61	-0.094
**Pd10**	3.0	0.57/0.61	0.186	3.0	0.68/0.59	-0.154
**Mulitlocus**	3.6	0.59/0.65	0.074	3.6	0.67/0.65	0.005

Sample size is 62 for the Santa Cruz and Isabela cluster and 76 for the Floreana cluster for all loci. A: allelic richness (estimated for a sample size of 75 individuals); Ho:observed heterozygosity; He: expected heterozygosity; Fis (inbreeding coefficients) were calculated after Weir and Cockerham [40]. None of the loci had a significant heterozygote deficiency or excess after sequential Bonferroni correction.

## Discussion

In combination with the microsatellite data, our mitochondrial findings are consistent with there being one species of *Philornis *on the islands from which we sampled. A population bottleneck was detected in the entire sample of individuals from the three islands, which is consistent with the pattern expected from an invasive, recently colonised species [4-6]. We report low genetic differentiation between island populations of the invasive avian parasite *P. downsi *on the Galápagos archipelago. Fly populations on Santa Cruz, Floreana, and Isabela showed strong evidence for high inter-island gene flow. However, low levels of divergence were detected between individuals from Floreana Island and those from Santa Cruz and Isabela when incorporating geographic sampling information. The molecular variance was mainly explained at the level of individuals, and not by island, which further demonstrates the low genetic differentiation between islands. Bayesian clustering analysis with geographic data assigned individuals to two genetic clusters, one comprising individuals from Santa Cruz and Isabela, and the second comprising all individuals from Floreana Island (Table 3, Figure 4). This might indicate that gene flow in *P. downsi *between Floreana and the other islands is restricted to some extent, or that this island underwent a distinct founding process. Pairwise *F*st between the three islands further indicated that flies on Floreana may be slightly genetically divergent from flies on the other two islands.

The Bayesian clustering method implemented in STRUCTURE is considered to be best able to infer correct individual assignments when genetic differentiation between populations is well defined [48]. Furthermore, the ability to distinguish the source of an individual decreases under conditions of high dispersal and associated low genetic differentiation [51,55]. The level of genetic differentiation (*F*st) between populations is found to be a useful predictor of the performance of assignment methods [55]. In the current study, the inability of STRUCTURE to confidently assign individuals to any cluster with certainty may reflect the lack of power to do so due to the low genetic differentiation (i.e. *F*st) between sampling locations. Thus, we conclude there was an insufficient signal in the data to confidently assign individuals under the model of Pritchard et al. [48], despite reasonably high PI values across loci. Our results are therefore testament that taking the spatial context of individuals into account improved the efficiency of our analysis, as found by Fontaine et al. [53]. Verifying the usefulness of STRUCTURE to assign individuals correctly where genetic differentiation is low and dispersal is common requires further study using empirical field data [51,55].

The current study lacks genetic data from mainland *P. downsi *populations and data from all islands of the Galápagos where *P. downsi *occurs, which will be necessary for a detailed examination of founder effects, bottlenecks, introduction events and colonisation pathways. Thus, without knowing where *P. downsi *populations originally came from, or where they most recently arrived on the Galápagos archipelago, a comprehensive invasion history can not be constructed on a demographic or evolutionary scale [11,56]. However, our findings lay the foundation for a more thorough understanding of the process of *P. downsi *invasion on the Galápagos archipelago. It is possible that *P. downsi *arrived on Ecuadorian cargo ships that were transporting fruit to the islands for human consumption [57,58], while it is also suggested that the fly came with imported pigeons (discussed in [59]). Strong winds and air currents present during El Niño events on the Galápagos are believed to contribute to insect dispersal between islands [60], while transport of humans and materials is also suspected to aid inter-island insect dispersal. In four other invasive insect species, the dates of colonisation on each island suggest a wind-mediated southeast to northwest direction of colonisation across the islands [57]. Such patterns remain unexplored for *P. downsi*.

### Invasion processes

Recently colonised invaders are often subject to a reduction in genetic variation and population bottlenecks because populations are not in genetic equilibrium [4-6]. We provide evidence for a population bottleneck in *P. downsi *across the three islands examined, which could be due to a small founding population, low immigration rates, or few introduction events [6,61]. The low allelic diversity across loci and population bottleneck in *P. downsi *is further evidence for a small effective population size upon initial colonisation. However, the occurrence of multiple introductions can not be excluded, particularly in the absence of comparisons with potential source populations (e.g. from Ecuador, Trinidad, or Brazil) [13]. Despite the presence of a population bottleneck and the (most likely related) low genetic diversity in *P. downsi*, the fly has clearly succeeded at establishing and spreading itself across the archipelago in high numbers.

Recently established species may persist at low and possibly undetectable numbers before becoming noticeably abundant and invasive years or decades later [5], which may reflect the lag time (i.e. the time between arrival and spread) observed in many species that become invasive [62]. This scenario seems likely concerning the invasion of *P. downsi *on Galápagos because the fly was not detected in finch nests and identified until 1997 [63], despite the recent discovery of specimens found in collections made in 1964 [13,17]. The parasite has since spread successfully and in high numbers across the archipelago (11 of 13 major islands) [59], indicating that any lag period that took place has passed. Yet it is unknown how recently each island was colonised and thus, whether particular island populations are undergoing a lag period that would favour the success of an immediate eradication effort (discussed in [64]).

Ecological [12,28,59] findings do not support the current existence of a lag period and indicate that *P. downsi *has spread successfully in at least 12 avian host species on the Galápagos Islands [12,13]. In the current study, we provide evidence that the *P. downsi *population on Floreana Island has detectable levels of genetic differentiation when compared with two other island populations, which might be the result of a separate introduction event(s) or colonisation pattern. A wider geographic sample of locations across habitats and islands is needed to examine this more definitively in combination with a larger number of highly polymorphic genetic markers. However, it is clear that *P. downsi *populations generally have high connectivity between islands or high shared ancestry, although variation in population processes (e.g. rates of dispersal, colonisation histories) between particular islands may allow for low levels of inter-island genetic differentiation.

### Absence of local genetic divergence

Local populations are expected to evolve adaptive differences in response to differing environmental conditions [64]. The lack of genetic structure in *P. downsi *on the Galápagos archipelago may reflect the estimated short time period since the flies' introduction (~40 years ago) [3] such that populations have not yet diverged since colonisation. We document no genetic structure according to habitat type across islands, which implies high levels of fly dispersal between the two habitats. Across islands however, differences in host diversity and distribution, ecological variables, or colonisation history may result in genetic divergence due to genetic drift, as was evident from the low genetic differentiation we document on Floreana Island.

Fly populations may show rapid evolution with geographic cline, as shown by Huey et al. [65] who found increased wing length with latitude in *Drosophila subobscura*, just two decades after its introduction into North America. The evidence we present for high gene flow between habitats implies that morphological variation in *P. downsi *is unlikely, though other insect species on Galápagos show morphological variation and genetic differentiation between habitats and islands of the archipelago [56,66,67]. Clinal variation in morphology (and evidence for low dispersal) was also found for *Bulimilus *land snails on Galápagos [68] and Darwin's small ground finch [26].

### Implications for control: the sterile insect technique (SIT)

The use of SIT to control *P. downsi *on the Galápagos Islands is perhaps the most appropriate method for eradicating an invasive fly within this ecologically fragile island ecosystem. SIT is a non-disruptive method as it does not introduce toxic or foreign chemicals into the environment, it is species specific, and does not introduce new genetic material into populations because the released organisms are not self-replicating [19,69].

The effectiveness of SIT is affected by population genetic differentiation within the target species because the occurrence of undetected sub-species or strain differentiation across geographic populations can be detrimental to widespread sterile male release [19]. Reinfestation of parasitic flies in SIT treated regions have been explained by genetic differentiation in the target species among allopatrically separated populations that may be experiencing reproductive isolation [e.g. [70]]. It is therefore of great advantage to use molecular genetic techniques for species characterisation and to examine population genetic structure prior to establishing large-scale sterile male release programs. We show that gene flow in *P. downsi *within and between three islands of the Galápagos is high, and unlikely to result in reproductive isolation. Thus, release of a single sterile strain of *P. downsi *could effectively suppress and eradicate the fly across the archipelago. Captive breeding experiments of adult *P. downsi *from multiple island populations are necessary to determine this with high confidence.

## Conclusion

The wide habitat range and high dispersal capacity of *P. downsi *highlights the significant threat that this parasite poses to the Galápagos endemic avifauna. Our findings are concordant with the prediction that parasites with low host specificity [28], good dispersal ability and horizontal transmission will show low population genetic structure and differentiation [56]. Ideally, it is best to eradicate invasive species before they become adapted to the local environment in which they have colonised and prior to repeated invasions with the aid of strict quarantine practices [7]. For *P. downsi*, this window of opportunity appears to have passed, prompting the need for a long-term eradication program combined with sustained quarantine and monitoring practices.

## Authors' contributions

RYD and MGG performed all molecular laboratory work. RYD performed all genotyping, data analysis and drafted the manuscript. MGG and SD participated in data analysis and interpretation. SK participated in the design and coordination of the study and provided funding. SK and RYD conducted field work and collection of samples. All authors read and approved the final manuscript.
